# Oleic Acid Protects Against Insulin Resistance by Regulating the Genes Related to the PI3K Signaling Pathway

**DOI:** 10.3390/jcm9082615

**Published:** 2020-08-12

**Authors:** Carlos López-Gómez, Concepción Santiago-Fernández, Sara García-Serrano, Eva García-Escobar, Carolina Gutiérrez-Repiso, Cristina Rodríguez-Díaz, Ailec Ho-Plágaro, Flores Martín-Reyes, Lourdes Garrido-Sánchez, Sergio Valdés, Alberto Rodríguez-Cañete, Francisca Rodríguez-Pacheco, Eduardo García-Fuentes

**Affiliations:** 1 Unidad de Gestión Clínica de Aparato Digestivo, Hospital Universitario Virgen de la Victoria/Instituto de Investigación Biomédica de Málaga-IBIMA, 29010 Málaga, Spain; carlos.lopez@ibima.eu (C.L.-G.); conchisantiagofernandez@gmail.com (C.S.-F.); cris.rdrz@gmail.com (C.R.-D.); ailec_hp@hotmail.com (A.H.-P.); floresmarey@hotmail.com (F.M.-R.); 2 Unidad de Gestión Clínica de Endocrinología y Nutrición, Hospital Regional Universitario de Málaga/Instituto de Investigación Biomédica de Málaga-IBIMA, 29010 Málaga, Spain; garciasara79@hotmail.com (S.G.-S.); eyring@gmail.com (E.G.-E.); sergio.valdes@hotmail.es (S.V.); 3 CIBER de Diabetes y Enfermedades Metabólicas Asociadas-CIBERDEM, 29010 Málaga, Spain; 4 Unidad de Gestión Clínica de Endocrinología y Nutrición, Hospital Universitario Virgen de la Victoria/Instituto de Investigación Biomédica de Málaga-IBIMA, 29010 Málaga, Spain; carogure@hotmail.com (C.G.-R.); lourgarrido@gmail.com (L.G.-S.); 5 CIBER Fisiopatología de la Obesidad y Nutrición-CIBEROBN, 29010 Málaga, Spain; 6 Unidad de Gestión Clínica de Cirugía General, Digestiva y Trasplantes, Hospital Regional Universitario de Málaga, 29010 Málaga, Spain; arodriguezcane@hotmail.com

**Keywords:** morbid obesity, insulin resistance, IRS1, p85α, p110β, palmitic acid, oleic acid, docosahexaenoic acid, linoleic acid, visceral adipose tissue

## Abstract

Background: The effects of different types of fatty acids on the gene expression of key players in the IRS1/PI3K signaling pathway have been poorly studied. Material and Methods: We analyzed IRS1, p85α, and p110β mRNA expression and the fatty acid composition of phospholipids in visceral adipose tissue from patients with morbid obesity and from non-obese patients. Moreover, we analyzed the expression of those genes in visceral adipocytes incubated with oleic, linoleic, palmitic and dosahexaenoic acids. Results: We found a reduced IRS1 expression in patients with morbid obesity, independent of insulin resistance, and a reduced p110β expression in those with lower insulin resistance. A positive correlation was found between p85α and stearic acid, and between IRS1 and p110β with palmitic and dosahexaenoic acid. In contrast, a negative correlation was found between p85α and oleic acid, and between IRS1 and p110β with linoleic, arachidonic and adrenic acid. Incubation with palmitic acid decreased IRS1 expression. p85α was down-regulated after incubation with oleic and dosahexaenoic acid and up-regulated with palmitic acid. p110β expression was increased and decreased after incubation with oleic and palmitic acid, respectively. The ratio p85α/p110β was decreased by oleic and dosahexaenoic acid and increased by palmitic acid. Conclusions: Our in vitro results suggest a detrimental role of palmitic acid on the expression of gene related to insulin signaling pathway, with oleic acid being the one with the higher and more beneficial effects. DHA had a slight beneficial effect. Fatty acid-induced regulation of genes related to the IRS1/PI3K pathway may be a novel mechanism by which fatty acids regulate insulin sensitivity in visceral adipocytes.

## 1. Introduction

Insulin resistance (IR) is a common complication in obesity and leads to the development of the metabolic syndrome and Type 2 Diabetes Mellitus (T2DM). IR is characterized by an abnormally reduced cell response to insulin signaling in target cells, such as adipocytes, hepatocytes, and skeletal muscle. After the binding of insulin with its receptor [1], docking proteins insulin receptor substrate (IRS) 1, 2, 3, and 4, as well as several Shc proteins, are mobilized to the intracellular domain of the insulin receptor. IRS1 and IRS2 are strongly expressed in insulin-sensitive tissues [2] and are responsible for many of the metabolic effects of insulin through the activation of phosphatidylinositol 3 kinase (PI3K). Mice with a targeted disruption of the IRS1 gene show either insulin resistance [3] or T2DM [4].

PI3K exists as a heterodimer composed of a catalytic subunit associated with a regulatory subunit. Among those subunits, p85α (regulatory) and p110β (catalytic) are some of the most studied ones. There are two widely distributed isoforms of the catalytic subunit, p110α and p110β, the latter being highly insulin-sensitive [5]. In fact, two mechanisms involving this pathway have emerged as being mainly responsible for the development of IR: Serine phosphorylation of IRS1 [6,7], and increased expression of p85α and alteration of the stoichiometry with p110β. Both the pool of free p85α subunit and the heterodimer p85α/p110β compete for the binding sites of IRS proteins. Thus, increased expression of p85α leads to decreased PI3K activity, subsequently decreasing the sensitization to insulin [8]. P85 expression has been found to be increased in insulin-resistant human skeletal muscle and could represent an important cellular mechanism contributing to skeletal muscle insulin resistance in obesity and T2DM [7].

The association between fatty acids and insulin resistance is widely known. Its level in serum is elevated in most patients with morbid obesity, [9] with a positive association with insulin resistance [10]. Several mechanisms, such as a defect in insulin-stimulated glucose transport caused by a defect in insulin signaling [11], activation of PKC and JNK kinases, activation of TLR4 and mitochondrial dysfunction have been described to explain, in part, this association [12]. The association between dietary lipids, cell membrane phospholipid composition and insulin resistance is also known [13,14,15,16]. An inverse association was found between insulin resistance and the level of different fatty acids in the erythrocyte membrane [14]. However, elevated fatty acids levels are not always necessarily associated with insulin resistance [17]. Among other causes, this could be due to a different composition of these fatty acids [9]. Different types of fatty acids can have opposite effects, as in the case of palmitic and oleic acids [18]. Also, the involvement of different fatty acids in the IRS/PI3K signaling cascade has not been sufficiently investigated. One study found that palmitate had no effect on p85α protein expression or on the activity of p110β [19] in myotubes. Instead, the authors found that palmitate inhibited the phosphorylation of Akt by reducing the phosphorylation of Src. In contrast, a different study found that palmitate does not alter gene expression of the insulin receptor and IRS1, but reduced its protein levels by inducing ubiquitination and proteasomal degradation of both proteins [20], and reduced the insulin-stimulated IRS-1 tyrosine phosphorylation in the myotubes from lean subjects [21].

To our knowledge, there are few studies investigating the effects of different types of fatty acids, such as palmitic (saturated), oleic (monounsaturated), linoleic (n-6 polyunsaturated) or dosahexaenoic (DHA) (n-3 polyunsaturated) acids on the gene expression of key players in the IRS1/PI3K signaling pathway in adipocytes. In the present study, we investigated the association of different fatty acids with the expression of the genes involved in this signaling cascade.

## 2. Material and Methods

### 2.1. Subjects

We selected 18 healthy, non-obese (NO) patients (BMI < 25 kg/m^2^) and 47 patients with morbid obesity (BMI > 40 kg/m^2^), 14 with low IR (homeostasis model assessment of insulin resistance (HOMA-IR) level < 4.7) (MO-low-IR), 24 with high IR (HOMA-IR > 4.7) (MO-high-IR) (both groups without treatment for T2DM) and 9 with T2DM under treatment with metformin (MO-metf-T2DM). The characteristics of these patients are summarized in Table 1. All patients with morbid obesity underwent laparoscopic Roux-en-Y gastric by-pass (RYGB). Subjects were excluded if they had T2DM and were receiving insulin treatment or other oral hypoglycemic medications, had cardiovascular disease, arthritis, acute inflammatory disease or infectious disease. The weight of patients with morbid obesity had been stable for at least one month before bariatric surgery. The non-obese patients underwent laparoscopic surgery for hiatus hernia or cholelithiasis, had no alterations in lipid or glucose metabolism, were of a similar age and with the same selection criteria as the patients with morbid obesity. All patients (non-obese and morbid obesity) were of Caucasian origin. Samples from subjects were processed and frozen immediately after their reception in the Regional University Hospital Biobank (Andalusian Public Health System Biobank). All the participants gave their written informed consent and the study was reviewed and approved by the Ethics and Research Committee of Regional University Hospital, Malaga, Spain (PS09/01060).

### 2.2. Biochemical Measurements

Blood samples from all subjects were collected before surgery and after a 12-h fast. The serum was separated and immediately frozen at −80 °C. Serum biochemical variables were measured in duplicate. Serum glucose, cholesterol and triglycerides (Randox Laboratories Ltd., Antrium, UK) were measured by standard enzymatic methods. Adiponectin levels were measured by enzyme immunoassay (ELISA) kits (DRG Diagnostics, Marburg, Germany). Leptin levels were measured by ELISA kit from Mediagnost (Reutlingen, Germany). The insulin was analyzed by an immunoradiometric assay (BioSource International, Camarillo, CA, USA). HOMA-IR was calculated with the following equation: HOMA-IR = fasting insulin (µIU/mL) × fasting glucose (mmol/L)/22.5 [22].

### 2.3. Adipose Tissue Sample Collection

Visceral adipose tissue samples from the omentum (VAT) were obtained during laparoscopic RYGB in the patients with morbid obesity (*n* = 47) and during laparoscopic surgery for hiatus hernia in the non-obese patients (*n* = 18) [23,24]. The biopsy samples were washed in physiological saline and immediately frozen in liquid nitrogen. Biopsy samples were maintained at −80 °C until analysis. Additional VAT samples from MO-low-IR (*n* = 6) were placed in phosphate-buffered saline (PBS) supplemented with 5% bovine serum albumin (BSA) to perform the adipocyte isolation and in vitro incubations. We chose to carry out this experiment in this group of patients to check if different types of fatty acids could reverse the findings observed in the VAT of this group of patients. These patients had the same biochemical and anthropometric characteristics as the MO-low-IR group.

### 2.4. Fatty Acid Composition of Phospholipids from Visceral Adipose Tissue

Total lipids from frozen VAT samples were extracted with chloroform-methanol 2:1 (*v*/*v*). The phospholipids were separated by thin-layer chromatography on silica gel plates (Merck, Darmstadt, Germany) with hexane-ethylic ether-acetic acid (80:20:2, *v*/*v*/*v*) as the developing solvent. Phospholipids were analyzed as described [9].

### 2.5. Adipocyte Culture and Fatty Acid Treatment

VAT adipocytes were isolated by digesting freshly isolated VAT with 1 mg/mL collagenase (Worthington Biochemical Corporation, Lakewood, NJ, USA) in Dulbecco’s Modified Eagle Medium (DMEM) for 1 h at 37 °C in a shaking water bath. Digests were filtered and centrifuged at 300× *g* for 10 min. Adipocytes were washed twice with DMEM and cultured in 24-well plates (50.000 adipocyte/wells) with DMEM (4.5 g/L glucose) supplemented with 10% BSA fatty acids free, 1% L-glutamine, and 1% penicillin/streptomycin for 24 h. Adipocytes were incubated with either 0.1 M NaOH (vehicle), oleic, linoleic, palmitic and DHA. Incubations with fatty acids were carried out at 25, 50 and 100 µM [25,26]. All four fatty acids were from Sigma-Aldrich (St. Louis, MO, USA). To prepare stock solutions, each of them was dissolved in 0.1 M NaOH at 80–90 °C. The resulting stock solutions were added to calcium-free DMEM containing 0.5% BSA-fatty acid-free to treat the isolated adipocytes. Each treatment was performed in triplicate. Following these treatments with fatty acids for 24 h, adipocytes were collected for mRNA isolation.

### 2.6. RNA Isolation and Quantitative RT-PCR

Total RNA from adipose tissue and adipocytes was extracted by RNeasy lipid tissue mini kit (Qiagen Science, Hilden, Germany) following the instructions of the manufacturer. cDNA was synthesized by retrotranscription using the M-MLV retrotranscriptase. Gene expression levels were analyzed in triplicate by quantitative real-time RT-PCR using a RotorGene Q Real-Time PCR system (Qiagen Science, Hilden, Germany), as previously described [27]. The primers used were designed with the online program Primer3 (http://frodo.wi.mit.edu/cgi-bin/primer3/primer3_www.cgi): IRS1 (NM_005544.2, forward: 5’-caagaccatcagcttcgtga-3´, reverse: 5´-agagtcatccacctgcatcc-3´); p85α (NM_181524.1, forward: 5´-accccagtttttgttgcttg-3´, reverse: 5´-actgcccaacaaaaccgtcc-3´); p110β (NM_001256045.1, forward: 5´-ggatgttgccttatggctgt-3´, reverse: 5´-ccttggagatgctgaaaagc-3´); β-actin (NM_001101.5, Fwd: 5´-tacagcttcaccaccacggc-3´; Rev: 5´-aaggaaggctggaagagtgc-3´). Gene expression was assessed using the 2^-∆∆Ct^ method and β-actin as a reference gene.

### 2.7. Statistical Analysis

The statistical analysis was done with R statistical software, version 2.8.1 (Department of Statistics, University of Auckland, Auckland, New Zealand; http://www.r-project.org/). Differences in the mean between more than two groups were compared by the Kruskal–Wallis test. Differences between the two matched groups were analyzed by the Wilcoxon test. The Spearman correlation coefficients were calculated to estimate the associations between variables. Values were considered to be statistically significant when the *p* ≤ 0.05. The results are given as the mean ± standard deviation (SD) or mean ± standard error of the mean (SEM) in figures.

## 3. Results

### 3.1. Fatty Acid Composition of VAT Phospholipids

Levels of individual fatty acids from VAT phospholipids are detailed in Table 2. Compared to non-obese patients, MO-low-IR showed significantly lower levels of stearic and higher levels of vaccenic acids (*p* = 0.016 and *p* = 0.011, respectively). MO-high-IR also showed significantly higher levels of vaccenic acid (*p* = 0.036), as well as higher levels of arachidonic acid (*p* = 0.044). MO-metf-T2DM only showed significantly higher levels of arachidonic acid (*p* = 0.014).

### 3.2. IRS1, p85α and p110β Gene Expression

**Figure 1:**
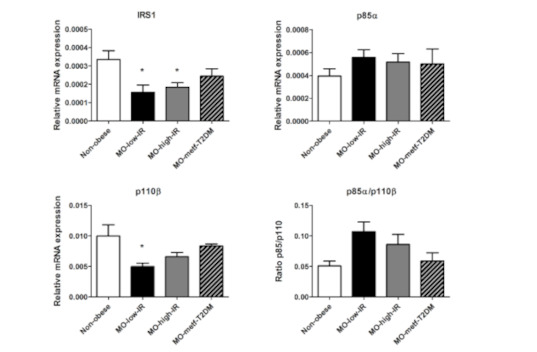
mRNA expression levels of IRS1, p85α, p110β and p85α/p110β ratio in VAT from non-obese patients and in patients with morbid obesity with low insulin resistance (MO-low-IR), with high insulin resistance (MO-high-IR), and with T2DM under treatment with metformin (MO-metf-T2DM). Gene expression was assessed using the 2^-∆∆Ct^ method. Results are represented as mean ± SEM. * *p* < 0.05 with regard to non-obese patients.

Analysis of the gene expression of key players of the IRS1/PI3K signaling cascade (Figure 1) showed a reduced expression of IRS1 in all three groups of patients with morbid obesity compared to non-obese patients, with only the MO-low-IR and MO-high-IR groups showing significant differences (*p* = 0.017 and *p* = 0.02, respectively). p110β was decreased in the three groups of morbidly obese patients, with only the MO-low-IR group showing significant differences (*p* = 0.036). There were no significant differences in p85α expression and p85/p110β ratio.

### 3.3. Association between VAT Phospholipid Composition and mRNA Expression

First, we wanted to know whether the levels of a particular fatty acid could be associated with the gene expression of IRS1, p85α or p110β, taking into account all patients (non-obese patients and those with morbid obesity) (Table 3). We found a significant positive correlation between p85α and both stearic and total saturated fatty acids. In contrast, p85α negatively correlated with oleic acid and total unsaturated fatty acids. Both IRS1 and p110β negatively correlated with the sum of all n-6 fatty acids, linoleic, arachidonic, and adrenic acid. In contrast, IRS1 and p110β positively correlated with palmitic and DHA. The ratio p85α/p110β was not correlated with any of the fatty acids assessed. However, no significant differences were found between gene expression of IRS1, p85α or p110β, and HOMA-IR and other biochemical variables described in Table 1.

### 3.4. In Vitro Response to Fatty Acids

**Figure 2:**
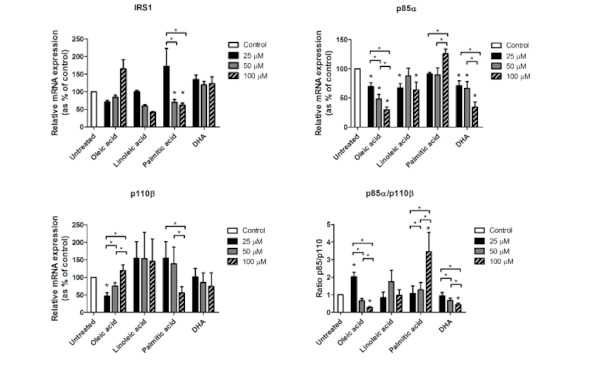
mRNA expression levels of IRS1, p85α, p110β and p85α/p110β ratio from isolated visceral adipocytes incubated with oleic, linoleic, palmitic and dosahexaenoic (DHA) acids. For each fatty acid, incubations were made with 25, 50 and 100 µM dose. Gene expression was assessed using the 2^-∆∆Ct^ method, which was converted as % of control (untreated). Results are represented as mean ± SEM. * *p* < 0.05 comparing each treatment and dose to the untreated sample (stars over bars) and between different doses inside each treatment group.

To assess a direct effect of these fatty acids on the gene expression of IRS1, p85α and p110β, we investigated in vitro the effects of the main fatty acids that were significant in the correlations shown above: Palmitic (saturated fatty acid), oleic (monounsaturated fatty acid), linoleic (n-6 polyunsaturated fatty acid), and DHA (n-3 polyunsaturated fatty acid). Accordingly, we incubated freshly isolated adipocytes from VAT during 24 h with each of these fatty acids at different concentrations (Figure 2). With regard to IRS1, only the incubation with palmitic acid decreased its expression compared to control (*p* = 0.043 for both 50 µM and 100 µM). Palmitic acid also decreased IRS1 expression in a dose-dependent manner (*p* = 0.043 between 25 µM and 50 µM, and *p* = 0.043 between 25 µM and 100 µM). Oleic acid increased IRS1 expression in a dose-dependent manner, but not significantly (*p* = 0.068 for tests between the three different doses). Linoleic acid decreased IRS1 expression in a dose-dependent manner, but not significantly (*p* = 0.068 for both 50 and 100 µM compared to the control, and *p* = 0.068 for tests between the three different doses).

With regard to p85α (Figure 2), oleic acid decreased its expression in a dose-dependent manner (*p* = 0.028 for all three doses with regard to the control; *p* = 0.028 between 25 µM and 50 µM; *p* = 0.028 between 25 µM and 100 µM; and *p* = 0.046 between 50 µM and 100 µM). Linoleic acid decreased p85α expression, but only at 25 µM (*p* = 0.012) and 100 µM (*p* = 0.018). DHA also decreased p85α expression in a dose-dependent manner (*p* = 0.025 for 25 µM, *p* = 0.036 for 50 µM and *p* = 0.012 for 100 µM with regard to the control; *p* = 0.017 between 25 µM and 100 µM; and *p* = 0.012 between 50 µM and 100 µM). Also, palmitic acid increased p85α expression with 100 µM with regard to 25 µM and 50 µM (*p* = 0.050 and *p* = 0.012, respectively).

Oleic acid also showed a dose-dependent effect on p110β expression (Figure 2), with the lower dose leading to significantly reduced levels of p110β (*p* = 0.018), but increasing significantly with higher doses (*p* = 0.018 for tests between the three different doses). 50 µM and 100 µM did not show significant differences with regard to the control. Also, 100 µM of palmitic acid significantly decreased p110β expression with regard to 25 µM and 50 µM (*p* = 0.043 and *p* = 0.043, respectively).

The p85α/p110β ratio (Figure 2) significantly increased with 25 µM of oleic acid (*p* = 0.043), but was further down-regulated with 50 µM and 100 µM doses (*p* = 0.043 for tests between the three different doses), reaching significantly reduced levels with regard to the control (*p* = 0.043). Similar to oleic acid, DHA also led to a dose-dependent down-regulation of p85α/p110β ratio (*p* = 0.043 for 100 µM with regard to the control; *p* = 0.043 between 25 µM and 50 µM; and *p* = 0.043 between 25 µM and 100 µM). The p85α/p110β ratio was dramatically increased with 100 µM of palmitic acid (*p* = 0.043 with regard to the control, and *p* = 0.043 for tests between the three different doses).

## 4. Discussion

The main result of this study was the different effect observed in the incubations with different types of fatty acids on the expression of genes involved in IRS1/PI3K pathway in visceral adipocytes. We observed a down-regulation of IRS1 and an increased p85α/p110β ratio with palmitic acid. However, oleic acid decreased p85α expression and the p85α/p110β ratio, and increased p110β. DHA had a moderate effect only on p85α expression and the p85α/p110β ratio, and linoleic acid was the one with the least effect in visceral adipocytes.

Obesity and insulin resistance/T2DM are associated with an imbalance in the PI3K/AKT signaling pathway [28]. As in previous studies [29,30], we found decreased levels of IRS1 VAT from patients with morbid obesity, independent of the insulin-resistant state, with metformin slightly increasing IRS1 expression. Different studies in human show that there is a decrease in IRS1 protein in the insulin-resistant human skeletal muscle [7], in obese muscle [31], and in adipose tissue of patients with T2DM [32]. Similar results were found in a study in the Zucker fatty rat model, in which insulin resistance was associated with a decreased levels of IRS proteins [33]. Also, our results showed a decrease of p110β and a slight increase, although not significant, of p85α expression and the p85α/p110β ratio, as in other studies in an insulin resistant state [7,34,35]. Our results with p110β agree with another study in which a decrease of p110 and IRS1 was observed in the muscle of insulin-resistant subjects [7]. However, elevated levels of protein p85 was a feature of insulin resistance in human skeletal muscle [7]. Cornier et al. also showed that three days of overfeeding (50% above usual caloric intake) significantly increased p85α, p85/p110 ratio and insulin resistance in human skeletal muscle [36]. Studies in animals with a disruption of p85α (p85^+/-^ heterozygous mice) showed a higher ratio of p85/p110 dimer to free p85 and were more sensitive to insulin [37].

High fatty acid levels have been commonly known to be associated with the development of IR [38]. However, not all individual fatty acids have the same effect. In fact, palmitic and oleic acid have been described as having the opposite roles in the development of IR [1]. Therefore, palmitic acid is known to induce IR through a variety of mechanisms [20,39]. Moreover, we demonstrated in a previous study that palmitic acid increases the inflammation markers in visceral adipocytes [26]. Palmitic acids also reduced the insulin-stimulated IRS-1 tyrosine phosphorylation in the myotubes from lean subjects [21], and increased insulin resistance in C2C12 skeletal muscle cells [40]. In contrast, oleic acid has been described to protect from IR [41]. In rodent muscle, oleic acid promoted GLUT4 through the PI3K pathway, preventing the effects of palmitic acid [42]. There are different mechanisms by which oleic acid prevents IR, such as reducing ER and mitochondrial stress, increasing mitochondrial β-oxidation and preventing inflammation [26,41,43,44].

However, the impact of each type of fatty acid (saturated, monounsaturated, n-6 and n-3 polyunsaturated) on the mRNA expression of IRS1 and PI3K subunits, which are key players in the metabolic response to insulin, have not been thoroughly assessed. To analyze that impact, we have analyzed the possible association between the fatty acid composition of VAT phospholipid with the expression of IRS1, p85α and p110β, and we further replicated the most relevant results in incubations of VAT adipocytes. Although the positive correlation between palmitic acid and IRS1 may contradict previous studies reporting the harmful effects of palmitic acid on IR, the dose-dependent down-regulation of IRS1 in response to palmitic acid observed in in vitro-cultured adipocytes suggests that IRS1 is down-regulated by this fatty acid. This result would agree with previous studies showing that palmitic acid induces IR [39]. The in vivo association may be affected by other factors not considered in this study, the final result of which could mask the relationship between palmitic acid and IRS1 expression. The downregulation of the in vivo IRS1 levels could be due to other molecules, such as TNFα, glucocorticoid and mineralocorticoid, which are increased in obesity [45,46,47,48]. We also found that other fatty acids positively correlated with IRS1 expression, such as oleic acid and DHA. However, neither of these two fatty acids produced a significant increase in the expression of IRS1, although this trend was observed with oleic acid. Regarding linoleic acid, its levels negatively correlated with IRS1 expression. Despite the lack of statistically significant results from in vitro-cultured adipocytes, we found a dose-dependent effect on IRS1 expression that was close to being significant. This suggests that this gene would, in fact, be down-regulated by linoleic acid, confirming the harmful effects of n-6 fatty acids on insulin sensitivity [49].

The increased expression of p85α has been suggested to be an early molecular step in the pathogenesis of nutritionally induced insulin resistance [1]. Previous reports have not observed any effects of palmitic acid on p85α protein expression [19]. Likewise, we did not observe a correlation between palmitic acid and p85α, although we observed an up-regulation at the higher dose used. In contrast, oleic acid negatively correlated with p85α, which was further confirmed in in vitro-cultured adipocytes, with a dose-dependent down-regulation of p85α. This down-regulation of p85α would increase the pool of p85α/p110β dimer available to bind to IRS1, therefore, increasing insulin sensitivity [1]. Therefore, our results suggest that oleic acid would have a beneficial effect in protecting from IR. DHA also down-regulated p85α, more dramatically at the higher dose. This effect was also observed in the correlation analysis, although it did not reach statistical significance. This down-regulation may also explain the protection of n-3 fatty acids to IR described in the literature [50]. 

The other PI3K subunit studied is p110β. Its expression in VAT positively correlated with palmitic, oleic and DHA. However, p110β expression in in vitro-cultured adipocytes only showed a significant increased in a dose-dependent manner with higher concentrations of oleic acid. Palmitic acid also positively correlated with p110β, although incubations showed a significant decrease of p110β expression with the higher dose. This discordance is similar to those found for IRS1. Specifically, correlations with palmitic acid could be affected by confounder factors not controlled in this study; alternatively, the effect of another fatty acid could prevail for the effects of palmitic acid. Further studies are necessary to confirm this hypothesis.

Together, the expressions of p85α and p110β could be affecting insulin signaling. p85α/p110β heterodimer is responsible for the PI3K activity. Although the p85α/p110β ratio did not initially correlate with any fatty acid, we observed different effects depending on the type of fatty acid. DHA and mainly oleic acid decreased the p85α/p110β ratio in a dose-dependent manner. A decrease of p85α/p110β ratio means lower free p85α to compete with the p85α/p110β monomer for the binding sites of IRS proteins. However, palmitic acid increased this ratio. An increase of p85α/p110β ratio [51] could cause an alteration of PI3K activity.

However, this study has several limitations. The effect of a single fatty acid does not represent the in vivo situation, which is a mixture of the effects of different types of fatty acids. Other studies use a coculture model with adipocytes and myotubes [52] to resemble in vivo physiology conditions. However, in our study, we wanted to separate the effects that each fatty acid may have on the expression of genes related to IRS1/PI3K pathways. Also, we have used this type of adipose tissue (VAT) because it is more associated with insulin resistance than other types of adipose tissue [53]. Another limitation refers to the type of subjects. Lipid metabolism can be highly dependent on ethnicity [54,55]. We limited our study to the Caucasian ethnicity because of lack of availability of samples from subjects from other ethnicities. Also, we obtained neither sufficient visceral adipocytes to measure the effects of other fatty acids that were differentially expressed between non-obese patients and those with morbid obesity, such as vaccenic and stearic acids, nor all the protein, phosphorylation levels and function of the genes studied. Adipocytes were only used for mRNA isolation. We think that the genes studied may be the most interesting ones involved in IRS1/PI3K pathways. However, other proteins are also involved, and it would have been interesting to study the effect of these fatty acids on their expression. Further studies are needed to analyze this and other aspects.

In conclusion, although changes in gene expression do not necessarily equate to changes in protein content or function, fatty acid-induced regulation of the genes involved in the IRS1/PI3K pathway may be a novel mechanism by which fatty acids regulate insulin sensitivity in visceral adipocytes. As expected, the impact of each fatty acid on the expression of key components of the IRS1/PI3K pathway is different. Most of the metabolic responses to insulin depend upon the activation of the IRS1/PI3K pathway. Overall, our in vitro results suggest a detrimental role of palmitic acid on the expression of gene related to insulin signaling pathway, being oleic acid the one with the higher and more beneficial effects. This effect of oleic acid subsequently would enhance insulin sensitivity. DHA showed a moderately beneficial effect, and linoleic acid was the one with the least effect on the insulin signaling pathway in visceral adipocytes. Further studies are needed to analyze other genes not studied in this work or to study the effects of each fatty acid on different types of adipose tissue and other tissues involved in the regulation of insulin resistance, such as muscle and liver. This will provide further evidence of the role of these fatty acids on the insulin signaling pathway. Also, it would be interesting to analyze the fatty acids composition of other lipids, such as diacylglycerides and acylcarnitines, which could be involved in the regulation of insulin resistance.

## Figures and Tables

**Table 1:** Anthropometric and biochemical variables in patients included in the study.

	Non-Obese	MO-Low-IR	MO-High-IR	MO-Metf-T2DM
*N* (men/women)	18 (6/12)	14 (5/9)	24 (9/15)	9 (3/6)
Age (years)	50.9 ± 16.6	47.8 ± 9.5	40.2 ± 8.4 ^a^	41.3 ± 12.1
Weight (kg)	74.6 ± 11.9	140.1 ± 29.1 ^a^	157.7 ± 27.2 ^a^	141.8 ± 22.4 ^a^
BMI (kg/m^2^)	27.6 ± 3.6	53.4 ± 6.4 ^a^	56.2 ± 6.6 ^a^	53.1 ± 6.0 ^a^
Glucose (mg/dl)	108.0 ± 24.3	97.0 ± 10.0	112.9 ± 36.5	169.1 ± 59.0 ^c^
Cholesterol (mg/dl)	190.9 ± 40.2	199.6 ± 42.9	196.7 ± 33.5	200.5 ± 28.9
Triglycerides (mg/dl)	79.4 ± 43.3	109.9 ± 39.7	175.6 ± 171.2 ^a^	172.0 ± 67.5
Insulin (μIU/mL)	10.8 ± 2.5	14.5 ± 1.8	29.2 ± 9.3 ^b^	30.7 ± 15.1 ^b^
HOMA-IR	2.9 ± 1.0	3.4 ± 0.5	8.0 ± 3.2 ^b^	13.7 ± 10.1 ^c^
Adiponectin (μg/mL)	20.6 ± 8.2	12.2 ± 4.7 ^a^	7.9 ± 4.1 ^a^	7.8 ± 4.3 ^a^
Leptin (ng/mL)	10.6 ± 3.2	183.2 ± 98.2 ^a^	151.7 ± 83.3 ^a^	168.2 ± 65.5 ^a^

The results are given as mean ± SD. ^a^ *p* < 0.05 significant differences with regard to non-obese group. ^b^ *p* < 0.05 significant differences with regard to non-obese and MO-low-IR. ^c^ *p* < 0.05 significant differences with regard to non-obese, MO-low-IR and MO-high-IR.

**Table 2:** Fatty acid composition of phospholipids in visceral adipose tissue from non-obese patients and morbid obese patients.

Fatty Acid		Non-Obese	MO-Low-IR	MO-High-IR	MO-Metf-TDM2
Lauric acid	12:0	0.58 ± 0.48	0.11 ± 0.17	0.25 ± 0.38	0.26 ± 0.24
Myristic acid	14:0	1.44 ± 0.29	2.18 ± 1.78	1.28 ± 0.92	1.40 ± 1.10
Palmitic acid	16:0	22.772.8	22.2 ± 1.9	22.9 ± 3.1	21.7 ± 1.5
Palmitoleic acid	16:1n-7	2.63 ± 0.81	2.92 ± 1.07	3.01 ± 0.82	2.41 ± 0.49
Stearic acid	18:0	15.8 ± 3.9	12.2 ± 2.0 ^a^	12.8 ± 2.2	12.8 ± 1.2
Oleic acid	18:1n-9	30.4 ± 4.8	28.2 ± 3.5	27.9 ± 4.6	26.5 ± 5.5
Vaccenic acid	18:1n-7	0.94 ± 1.33	2.62 ± 0.55 ^a^	2.35 ± 1.38 ^a^	2.10 ± 1.04
Linoleic acid	18:2n-6	16.1 ± 4.5	17.8 ± 2.0	14.7 ± 3.5	16.4 ± 3.0
Gamma-linolenic acid	18:3n-6	0.03 ± 0.08	0.02 ± 0.06	0.07 ± 0.27	0.01 ± 0.01
α-Linoleic acid	18:3n-3	0.29 ± 0.32	0.54 ± 0.84	0.28 ± 0.38	0.55 ± 0.54
Eicosanoic acid	20:0	1.01 ± 0.50	0.54 ± 0.54	1.09 ± 1.06	1.29 ± 0.51
Gondoic acid	20:1n-9	0.80 ± 0.61	0.89 ± 0.34	1.12 ± 1.79	1.13 ± 0.58
Eicosadienoic acid	20:2n-6	0.47 ± 0.51	0.73 ± 0.59	0.62 ± 1.06	0.87 ± 0.60
Dihomo-gamma-linolenic acid	20:3n-6	0.73 ± 0.34	1.17 ± 0.57	1.32 ± 1.33	1.79 ± 0.86
Arachidonic acid	20:4n-6	4.18 ± 1.48	5.62 ± 1.39	6.33 ± 1.99 ^a^	7.24 ± 2.28 ^a^
Eicosatrienoic acid	20:3n-3	0.05 ± 0.09	0.14 ± 0.28	0.33 ± 0.24	0.18 ± 0.20
Eicosapentaenoic acid	20:5n-3	0.35 ± 0.29	0.27 ± 0.32	0.50 ± 0.63	1.04 ± 1.49
Adrenic acid	22:4n-6	0.37 ± 0.35	0.72 ± 0.55	1.01 ± 1.03	0.68 ± 0.47
Docosapentaenoic acid	22:5n-3	0.24 ± 0.27	0.23 ± 0.35	0.65 ± 0.86	0.49 ± 0.48
Docosahexaenoic acid	22:6n-3	0.61 ± 0.98	0.80 ± 0.64	0.77 ± 1.01	1.08 ± 0.74
Total saturated fatty acids		41.6 ± 5.9	37.2 ± 3.7	38.9 ± 5.0	37.4 ± 2.3
Total monounsaturated fatty acids		34.7 ± 4.8	34.6 ± 4.0	34.4 ± 5.1	32.2 ± 5.5
n-3 polyunsaturated fatty acids		1.54 ± 1.45	1.99 ± 1.28	2.54 ± 2.34	3.35 ± 3.11
n-6 polyunsaturated fatty acids		22.0 ± 5.1	26.0 ± 2.1	24.1 ± 4.9	26.9 ± 3.7

Results are represented as mean ± SD. ^a^ *p* < 0.05 significant differences with regard to the non-obese patients.

**Table 3:** Significant correlations (*p*) between the fatty acid composition of phospholipids and mRNA expression of IRS1, p85α, p110β and p85α/p110β ratio in visceral adipose tissue.

Fatty Acid		IRS1	p85α	p110β	p85/p110β
Palmitic acid	16:0	*r* = 0.353	*r* = 0.234	*r* = 0.372	*r* = 0.033
		*p* = 0.017	*p* = 0.126	*p* = 0.012	*p* = 0.833
Stearic acid	18:0	*r* = 0.103	*r* = 0.484	*r* = −0.179	*r* = −0.152
		*p* = 0.501	*p* = 0.001	*p* = 0.240	*p* = 0.329
Oleic acid	18:1n-9	*r* = 0.284	*r* = −0.364	*r* = 0.303	*r* = 0.294
		*p* = 0.059	*p* = 0.015	*p* = 0.043	*p* = 0.056
Linoleic acid	18:2n-6	*r* = −0.416	*r* = −0.246	*r* = −0.348	*r* = 0.048
		*p* = 0.004	*p* = 0.108	*p* = 0.019	*p* = 0.762
Arachidonic acid	20:4n-6	*r* = −0.296	*r* = −0.123	*r* = −0.310	*r* = −0.063
		*p* = 0.049	*p* = 0.426	*p* = 0.038	*p* = 0.687
Adrenic acid	22:4n-6	*r* = −0.305	*r* = −0.139	*r* = −0.171	*r* = −0.083
		*p* = 0.041	*p* = 0.367	*p* = 0.261	*p* = 0.599
Docosahexaenoic acid	22:6n-3	*r* = 0.335	*r* = −0.211	*r* = 0.543	*r* = 0.145
		*p* = 0.025	*p* = 0.170	*p* < 0.001	*p* = 0.354
Total saturated fatty acids		*r* = 0.221	*r* = 0.446	*r* = 0.044	*r* = −0.047
		*p* = 0.144	*p* = 0.002	*p* = 0.775	*p* = 0.764
Total unsaturated fatty acids		*r* = −0.221	*r* = −0.446	*r* = −0.044	*r* = 0.047
		*p* = 0.144	*p* = 0.002	*p* = 0.775	*p* = 0.764
n-6 polyunsaturated fatty acids		*r* = −0.587	*r* = −0.266	*r* = −0.497	*r* = −0.062
		*p* < 0.001	*p* = 0.081	*p* = 0.001	*p* = 0.691
